# Practical Use of Composite Materials Used in Military Aircraft

**DOI:** 10.3390/ma14174812

**Published:** 2021-08-25

**Authors:** Lucjan Setlak, Rafał Kowalik, Tomasz Lusiak

**Affiliations:** 1 Department of Avionics and Control Systems, Military University of Aviation, 08-521 Deblin, Poland; r.kowalik@law.mil.pl; 2 Department of Thermodynamics, Lublin University of Technology, 20-618 Lublin, Poland; t.lusiak@pollub.pl

**Keywords:** practical use, composite materials, ANSYS and SolidWorks environment, military aircraft

## Abstract

The article presents a comparative characterization of the structural materials (composites and metals) used in modern aviation structures, focusing on the airframe structure of the most modern aircraft (Airbus A-380, Boeing B-787, and JSF F-35). Selected design and operational problems were analysed, with particular emphasis on composites and light metals (aluminium). For this purpose, the Shore’s method was used for the analysis of the obtained strength results and the programming environment (ANSYS, SolidWorks) required to simulate the GLARE 3 2/1-04 composite. The focus was on highlighting the differences in the construction and modelling of these materials resulting from their various structures (isotropy and anisotropy), e.g., by analyzing the mechanics of metal destruction and comparing it with the composite material. In terms of solving the problems of finite element analysis FEM, tests have been carried out on two samples made of an aluminium alloy and a fiberglass composite. The focus was on highlighting the differences in the construction and modelling of these materials resulting from their various structures (isotropy and anisotropy), e.g., by analyzing the mechanics of metal destruction and comparing it with the composite material. On the basis of the obtained results, the preferred variant was selected, in terms of displacements, stresses, and deformations. In the final part of the work, based on the conducted literature analysis and the conducted research (analysis, simulations, and tests), significant observations and final conclusions, reflected in practical applications, were formulated.

## 1. Introduction 

In the currently evolving technical progress, one can increasingly see the use of composite materials, in the aspect of advanced design solutions of most modern aircraft (Airbus A-380, Boeing B-787, SJF F-35, UAV). Thanks to this, it has become possible to produce not only fragments but also the entire support structure of both unmanned aerial vehicles (UAVs) and civil aircraft (Airbus, Boeing) or the military aircraft of a leading manufacturer, Lockheed Martin, in the case of the 5th generation JSF (joint strike fighter) F-35. Composites used in aviation structures should be characterized by the following properties, i.e., primarily high mechanical, impact, and thermal resistance [1,2,3,4,5,6,7].

**Figure 1:**
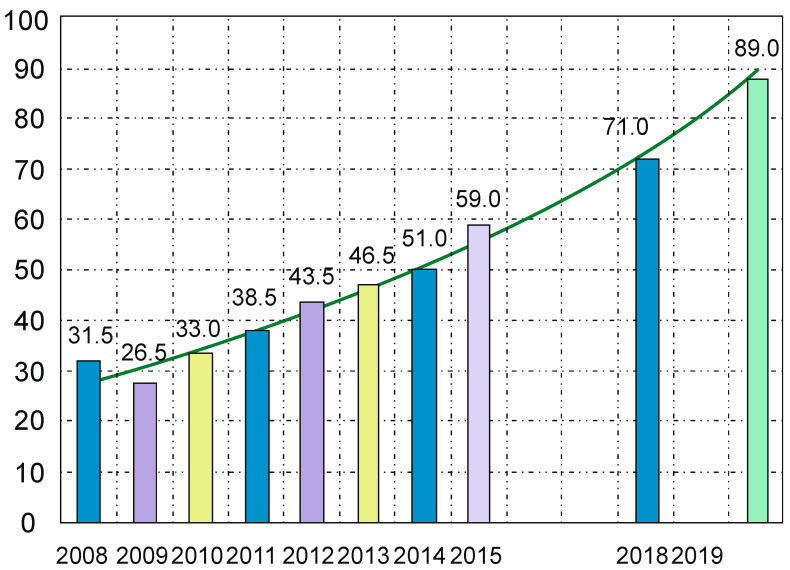
The development of the use of composites in recent years.

In recent years, there has been a noticeable dynamic increase in the implementation of composite materials in the aviation industry, which is shown in the diagram below (Figure 1) [8].

For example, during the production process for the V-22 Osprey [5] short take-off and landing aircraft, a design solution using composite materials and an airframe structure in the form of two spars and 18 ribs was created from graphite-epoxy composites. 

Whereas the flaperons are made of composites that rotate on the titanium mounting knots. Composites with epoxy matrix [1], reinforced with carbon fiber, are used in the design of the aircraft control system [9]. 

The conducted research has shown that it is possible to develop a new metallopolymer composite based on modified epoxy matrices. This composite is characterized by good strength properties, a low coefficient of friction, and high wear resistance. The resistance to the dynamic effects (impact strength) and compressive strength of the developed two-component metallopolymer composite is much higher, and its tribological properties are comparable to the regenerative composite produced and used so far.

Design solutions for the use of composite materials have also found application in military aviation, e.g., in the Lockheed C-5 transport aircraft [5]. It should be noted that the Lockheed C-5 aircraft is a structure in the high-wing configuration, using a composite-metallic structure. Its taiplane is made of composite-metal, while the wings of the airframe are made with a three-part structure (two-spar, metal) [10,11,12].

Another design solution using composite materials is the Lockheed Martin F-35 Lightning II multirole fighter aircraft [5], where composite materials take up 35% of the weight of the airframe. In the lifting elements, which include the wing sheathing, flaps, vertical and horizontal stabilizers, and the fuselage, a fiber-reinforced laminate and a polymer CFRP (carbon fiber reinforced polymer) cover were used.

It should be noted that this aircraft was a precursor in the mass production of aircraft, in which structural nanocomposites were used, referred to as epoxides reinforced with carbon nanotubes. The use of composites for this type of aircraft is also of strategic nature, as it allows for reduced detectability by radar stations [13,14,15,16,17].

Another example of the use of AFRP (aramid fiber reinforced polymer) composite materials is the American Boeing AH-64 Apache helicopter, in which composites were used to produce the main and tail rotor blades.

Aramid fibers, which are a component of composites, have been used, among others, in the design solutions of aircraft, yachts, space shuttles, brake lines, covers, and wherever a very high mechanical resistance of the material is required. Moreover, due to their very good fatigue strength, they are resistant to abrasion and have good electrical properties. These fibers do not undergo the process of melting and burning, undergoing only the process of carbonization at the temperature of 430 °C.

They are characterized by high thermal and mechanical strength, as well as impact resistance, which is extremely important in the construction of an aircraft [18]. With the passage of time, composite materials have found applications in many design solutions, mainly in aviation [5].

Other composite materials that have found applications in the aviation industry are ARALL (aramid aluminum laminates) and CARALL (carbon reinforced aluminum laminates) composites.

ARALL hybrid composite is a fatigue-resistant polymer composite, consisting of thin sheets of high-strength aluminum alloy in the range of 0.2–0.3 mm connected surface-to-face with aramid fibers. Their thickness is 0.22 mm, where the fibers share 50% of the structure by mass.

In turn, carbon fiber-reinforced aluminum laminates (CARALL) are one type of FML (fiber metal laminates) that are glued together to obtain a material with different properties than the original materials. As a result, it is a material sensitive to deformation. This type of composite is also a polymer composite, but reinforced with carbon fibers using epoxy resin, a composite layer of carbon fiber and aluminum.

For example, the design of the Swedish multirole Saab JAS 39 Gripen is based on the use of both metals and carbon fiber composites [19,20,21].

## 2. Description of the Tensile Test Stand, in Accordance with ASTM Standard

### 2.1. Research Description

In the research process, batches of selected composite samples were used, produced by the manual lamination method, with the use of epoxy resin (MGS L285/H285 type), characterized by the parameters presented in the table below (Table 1). The course of the study was limited to the use of fabric-based reinforcement in various orientations (0/+90° and +/−45°), then, at a further stage, a flexible bag was put on and fixed on the edges of the mold. The excess resin or air was extracted using a vacuum pump.

On the other hand, the laminate curing process was carried out at an atmospheric pressure of 0.9 atm. The next step was the preparation of subjecting the samples to the temperature equal to 60° C for 8 h. Then, samples of appropriate dimensions were cut out, in accordance with ASTM D3410 standard, from the resulting panel.

The final process of producing the samples used for the tests was sticking the overlays of the glass–epoxy composite on the edges of the laminate to protect the sample against damage, by fixing them in the holders of the testing machine.

Types of fabrics used for each series of the research process:

- A series—Dialead K63712 modular fabric;
- B series—Roving IMS65 (Intermediate Modulus carbon fiber);
- C series—IMS65 CTLX biaxial fabric;
- D series—symmetrical Interglas 02037 fabric;
- E series—modular fabric IMS65.

The ASTM D3039 standard is a test method that determines the in-plane tensile properties of high-modulus, fiber-reinforced, polymer-matrix composites. The purpose of the research method used was primarily to obtain parameter, in terms of stretching, data related to material specifications, and the ability to ensure good quality analysis.

The factors necessary during the research process include: methods of sample preparation, materials, geometric dimensions of samples, and the speed and time of testing. On the other hand, the values obtained through the tests, carried out in accordance with the applicable standard, were the key parameters, i.e., tensile strength, deformation, displacement, and the value of the force at maximum tension.

### 2.2. Microstructure of Composite Materials

Research in the field of the microstructure of composite materials was carried out with the use of specialized devices, in the form of microscopes. For this purpose, according to the research subject, the quality of the selected materials was carried out using the Olympus BX53M microscope (Oscyloskop Olympus Europa SE & Co. KG, Hamburg, Germany). 

#### 2.2.1. Description of the Test Stand

The new generation Olympus BX53M microscope has a Y-shaped base. Thanks to this, observations can be carried out in dark, bright, or mixed fields, as well as in fluorescence or reflected light.

The microscope stand was equipped with a replaceable turret handle, adjustable coarse screw rotation force, and a micrometric screw reading of min 1 µm. The lighting included a LED light source, with a power equivalent to 100 W in halogen lamps and a built-in preset for photography. It allowed for reproducible lighting conditions.

The possibility of observing the structure of materials, in the range of magnification from 12.5 to 500 times, as well as easy archiving of the measurements, made [22,23,24,25,26,27,28,29,30].

#### 2.2.2. Analysis of the Obtained Results

**Figure 2:**
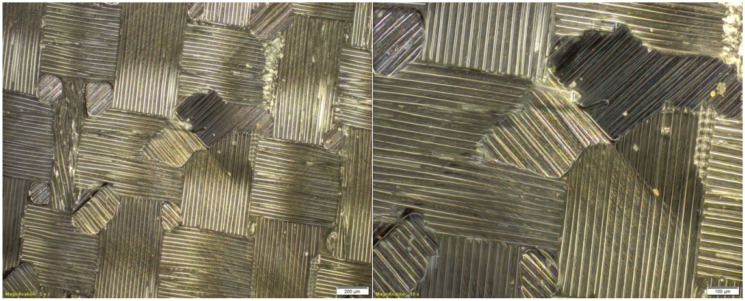
Microstructure of a composite laminate, reinforced with Dialead K63712 modular fabric, reprinted from [31].

A photo of the microstructure of the composite material, formed by combining Dialead K63712 modular fabric with MGS L285/H285 resin, is presented in the figure below (Figure 2). Some inclusions, in the form of visible particles, as well as air bubbles, can be observed in it, exerting an unfavourable influence on the composite structure.

**Figure 3:**
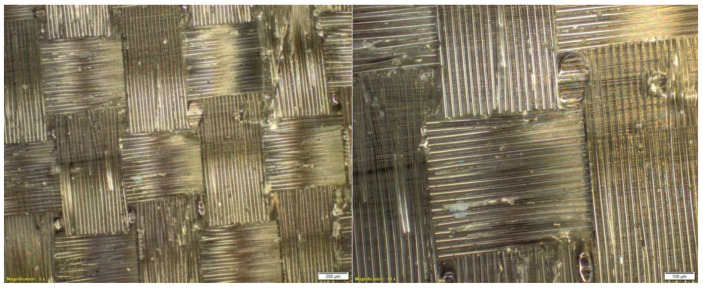
Microstructure of a composite laminate with Roving IMS65 reinforcement [32].

The study of the microstructure of the composite, with the use of Roving IMS65 reinforcement, allowed us to visualize the phenomenon of the appearance of air bubbles, which was shown in Figure 3.

**Figure 4:**
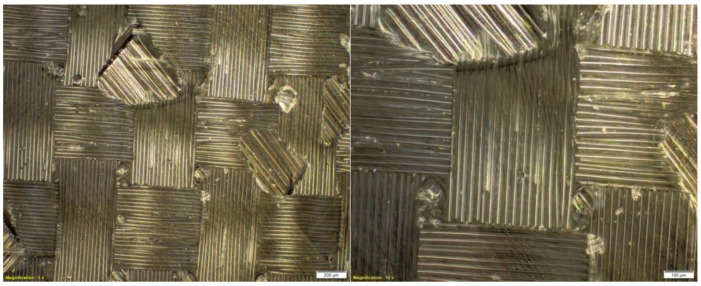
Microstructure of a composite laminate with the reinforcement of the IMS65 CTLX biaxial fabric, reprinted from [31].

On the basis of subsequent photos, obtained through microscopic examinations (Figure 4), it can be observed that in the examined area of the microstructure, numerous air bubbles are formed. They are the cause of the low strength of the composite material, which may result in the formation of preliminary microcracks in the structure of the composite laminate.

**Figure 5:**
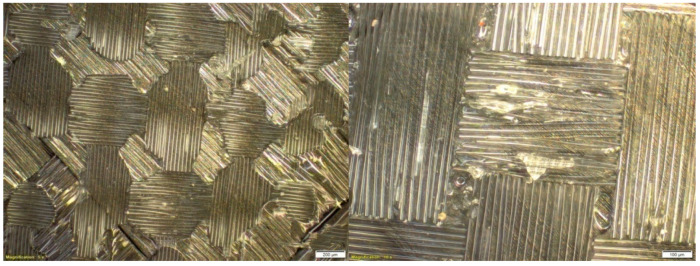
Microstructure of a composite laminate with the reinforcement of the symmetrical Interglas 02037 fabric [32].

The composite microstructure, presented in the figure below (Figure 5), shows that in the samples made, the phenomenon of delamination (i.e., delamination of fibers) occurs. A phenomenon of this type is disadvantageous because it indicates both damage to the material and a reduction in the cohesion of the layers; extensive air bubbles also appear.

**Figure 6:**
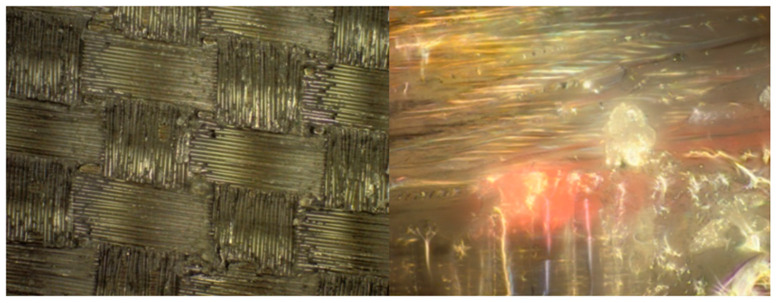
Microstructure of a composite laminate with the reinforcement of the IMS65 modular fabric [32].

The illustration of the next microstructure of composite samples (Figure 6), using the IMS65 modular fabric reinforcement with MGS L285/H285 matrix, shows a foreign body that could have penetrated into the composite structure during the manual lamination process. It should be noted that an emerging foreign body can reduce the strength of the composite, and a small amount of small air bubbles can also be seen.

### 2.3. Composite Laminate Hardness Testing 

Hardness is a feature of solids, which proves resistant to concentrated forces [33,34,35]. The Shore’s method, according to the standard PN-ISO 868, was used to determine the hardness and the indenter method, in accordance with the standard PN-93/C-04206. 

#### 2.3.1. Description of the Measuring Station 

**Figure 7:**
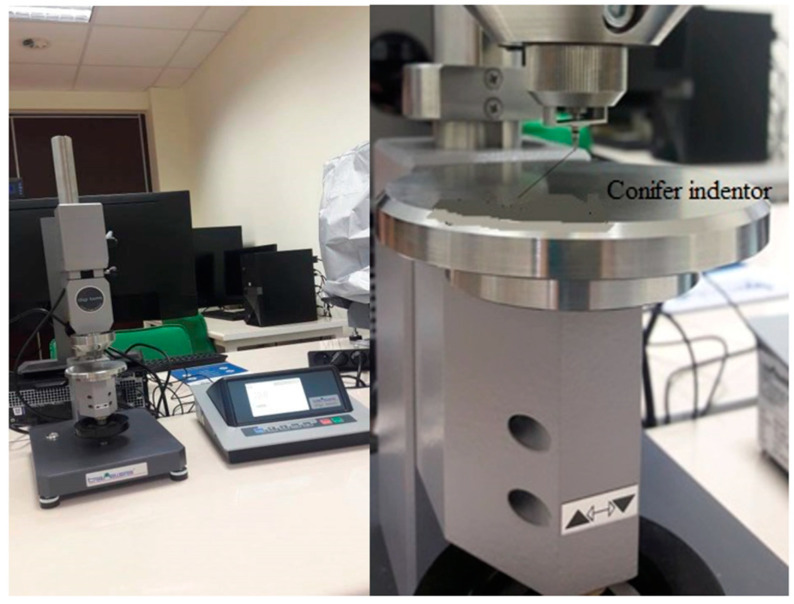
Shore’s hardness tester type M SHORE D/C/D0 0.5.

The measurement was carried out on a Shore’s durometer type M SHORE D/C/D0 0.5 (HILDEBRAND Prüf– und Meßtechnik GmbH, Oberboihinge, Germany) by pressing a pointed indenter harder than the tested material, which is illustrated in the next figure (Figure 7).

Technical Specifications:

- indication range: 0–100 Sh;
- pressure range within 1.961–490.3 N;
- load accuracy ±1%;
- measuring range: 20–90 Sh;
- load time 5–99 s (every 1s);
- mounting of interchangeable heads;
- resolution equal to: 0.1 or 0.5 Sh;
- magnification equal to 100×, 200×;
- maximum object height 210 mm;
- central unit with software.

#### 2.3.2. The Course of the Study and the Analysis of Measurements

**Figure 8:**
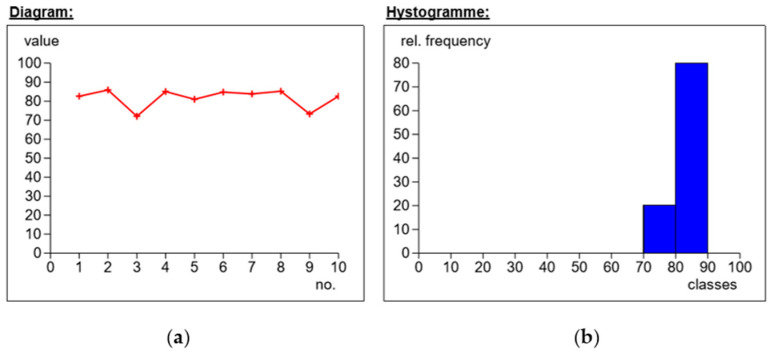
Application of Dialead K63712, weave 0/+90°, MGS L285/H285 warp: (**a**) dependence of hardness on the number of measurements; (**b**) percentages of hardness.

**Figure 9:**
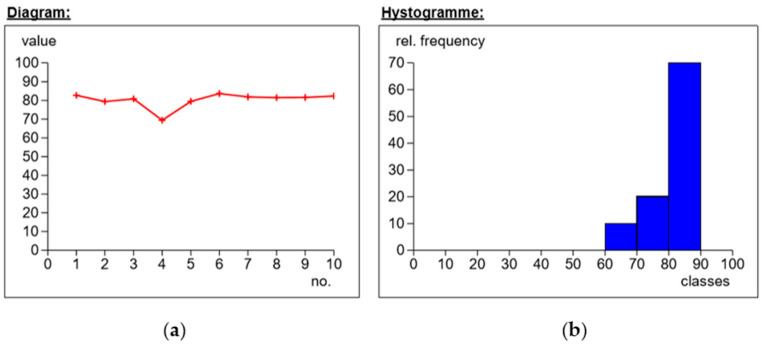
Use of Roving IMS65 fabric, weave 0/+ 90°, MGS L285/H285 warp: (**a**) dependence of hardness on the number of measurements; (**b**) percentages of hardness.

**Figure 10:**
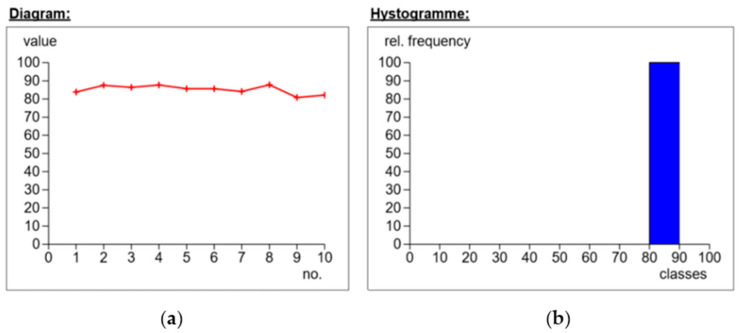
Application of IMS65 CTLX fabric, biaxial fabric, weave 0/+45°, MGS L285/H285 warp: (**a**) dependence of hardness on the number of measurements; (**b**) percentages of hardness.

**Figure 11:**
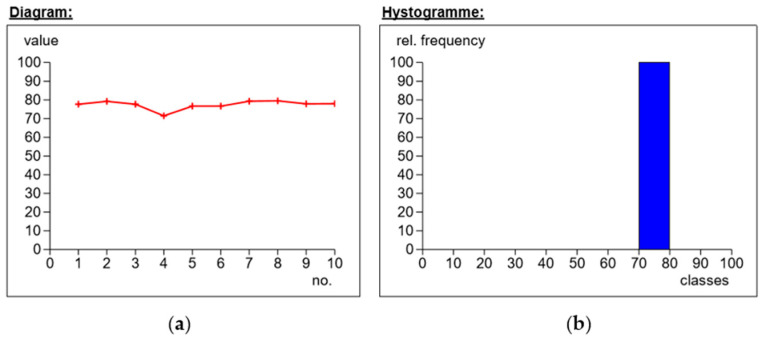
Application of modular fabric IMS65, weave 0/+ 90°, warp MGS L285/H285: (**a**) dependence of hardness on the number of measurements; (**b**) percentages of hardness.

First, the needle-shaped measurement indenter was mounted in the punch. The samples from each series were placed individually on the stage, and a rigid substrate was used to reduce shock or vibration. For one sample, 10 hardness measurements were carried out in different places on the surface of the tested material. The results of the obtained measurements are presented in the following figures (Figure 8, Figure 9, Figure 10 and Figure 11).

The time of the force (F) acting on the measuring indenter was 3s. The test was performed at room temperature, which was 19 °C, with a measurement range of 10 °C to 35 °C. During the tests, there were no disturbances caused by external factors, i.e., impacts or vibrations of samples with an indenter.

The obtained measurements (Figure 8) showed different hardness values for reinforcement with the use of the modular fabric Dialead K63712 weave 0/+ 90°. The highest measurement value was 85.8 Sh°D, the lowest 72 Sh°D, and the average value was 81.6 Sh°D.

On the basis of measurements 3 and 9, the phenomenon of delamination was found. Analyzing the histogram (subpoint b), the standard deviation was 4.96, with the differences in hardness values depending on the structural quality of the composites.

Based on the figure below (Figure 9), the values for measuring the hardness of the composite reinforced with Roving IMS65 fabric, with the use of plain weave, were obtained. The average value fluctuated around 80.2 Sh°D. The highest hardness was 83.6 Sh°D, while the lowest was 69.3 Sh°D. Based on the histogram, it was concluded that the standard deviation for the tested fabric was 4.

The next figure (Figure 10) shows the hardness measurement for the IMS65 CTLX fabric. The values fluctuated in the vicinity of 85 Sh°D. The highest value that was obtained was 87.7 Sh°D, and the lowest (measurement no. 9) was 80.7 Sh°D. By analyzing the histogram, it can be concluded that the standard deviation was equal to the value of 2.4. In turn, the different hardness results were due to the structural quality of the composite laminates.

The figure below (Figure 11) shows the measurements for the symmetrical Interglas 02037 fabric, weave 0/+90°, with a MGS L285/H285 warp. The results hovered around the value of 77.3 Sh°D. The maximum value was obtained in measurement 8 (i.e., 79.4 Sh°D) and the minimum in measurement 4 (i.e., 71.4 Sh°D). By analyzing the histogram, it can be concluded that the standard deviation was 2.3, one of the lowest values in the test of all series of composite laminate samples.

### 2.4. Tensile Test Stand

The samples were made in the shape of flat bars, in packages of several pieces of the same system and preparation; they were divided into 5 series, marked as follows:

XY, where:

X—letter of the alphabet from A to E defining a series that is equivalent to the type of the tested material;

Y—digit indicating the sample number from 1 to 9.

Then, the prepared samples were subjected to tension until destruction. The tests were carried out on the Instron testing machine, designed to perform static tensile tests of any materials, in various temperature ranges. They were carried out with a constant strain rate.

**Figure 12:**
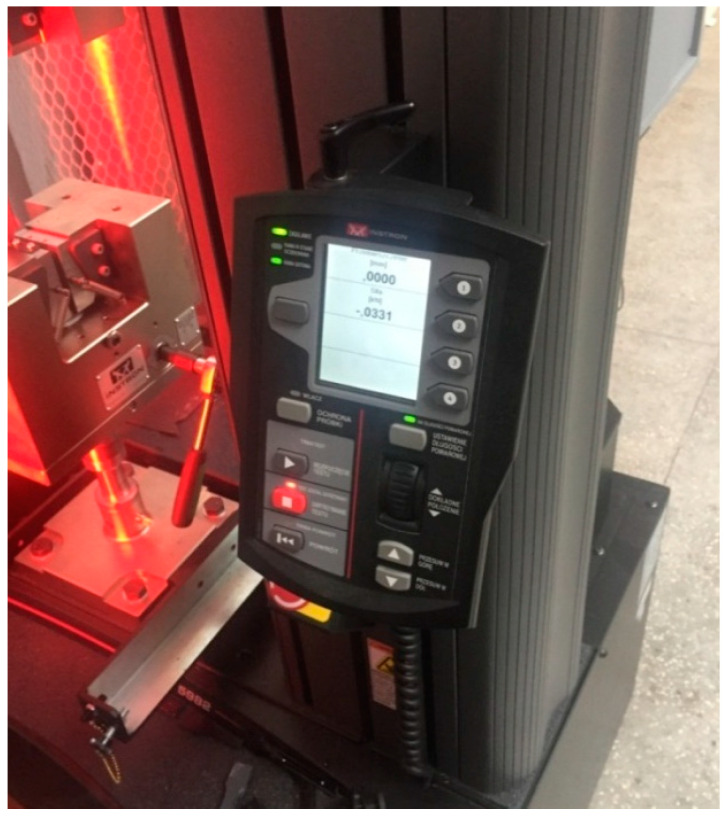
An example of setting the force parameters during the tensile test.

The data was sent directly from the measuring machine to a specialized program, where it was saved as a file. The obtained measurements, which were transmitted, can be adjusted by: time factor, change of force magnitude, or shortening/lengthening (Figure 12).

It should be noted that the significant advantages of the Instron testing machine were primarily the ease of use, through the automatic closing and opening of extensometers. Moreover, it is an alternative to determine the elastic modulus, an innovative control system that allows you to change parameters during tests and the possibility of having two working spaces.

In turn, the value of the force, recorded during the tests, with a very high accuracy for each of the ranges, did not exceed 5%.

### 2.5. Research Results and Analysis of the Results

The individual tables (Table 2, Table 3, Table 4, Table 5, Table 6, Table 7, Table 8 and Table 9) present the results of both the geometrical measurements of the samples used for the tests and their averaged values. The numbers of the illustrated samples in the charts have been assigned to the individual sample numbers in the given lot. The samples were made in accordance with the ASTM D3039 standard.

**Figure 13:**
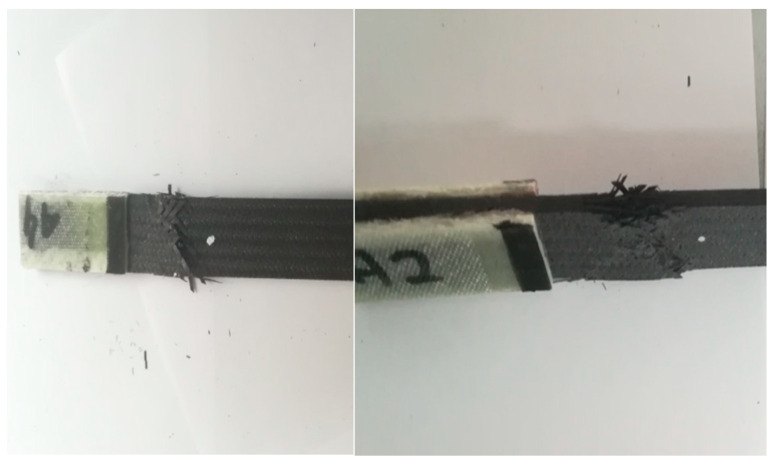
Appearance of samples from series A after tensile bench testing.

For the A series, reinforcement with the modular fabric Dialead K63712, with the use of epoxy resin marked MGS L285/H285, was used. During the strength test, a characteristic form of failure, of a given composite material, was obtained (Figure 13).

**Figure 14:**
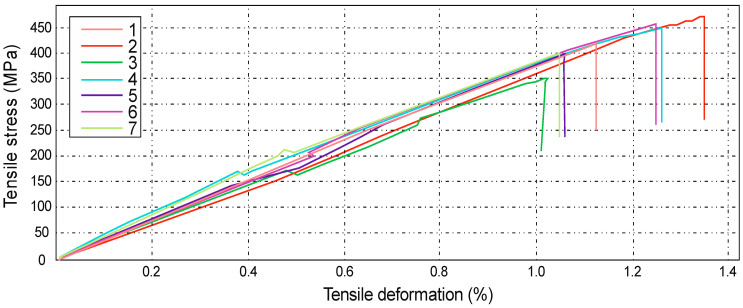
Graph showing the tensile stress and absolute deformation of a laminate reinforced with Dialead K63712 modular fabric, 0/90° configuration.

The figure below (Figure 14) shows the results obtained during the tensile test bench. The test was carried out for series A with specimens 1 to 7, consecutively marked. The maximum force during loading ranged from 23.03 kN to 30.55 kN. These values correspond to a tensile strength, ranging from 351 MPa to 468 MPa.

The greatest tensile elongation at break ranged from 1.03% to 1.35%, which corresponds to the displacement in the range from 3.59 mm to 5.29 mm. The result for the tensile strength of the composite laminate reinforced with Dialead K63712 modular fabric was equal to the value of $R_{m}$ = 419 ± 18 MPa.

For the B series, composite samples were made in accordance with the ASTM D3039 standard; the Roving IMS65 reinforcement was used with epoxy resin, designated MGS L285/H285. 

**Figure 15:**
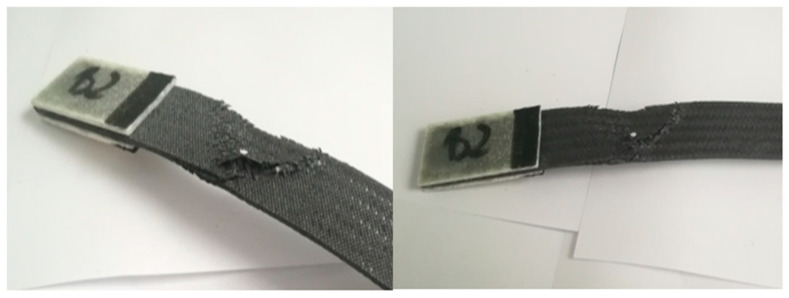
Appearance of samples from series B after tensile bench testing.

After the examination, a characteristic form of rupture and stretching of the fibers was obtained (Figure 15).

It should be noted that the shape of the samples must ensure an even stress distribution over the entire measured length. According to the guidelines, contained in the ASTM D3039 standard, samples were created, the dimensions of which are given in Table 2. 

Overlays with a lower stiffness than the tested material were glued to the edges of the composite.

The tensile strength test was performed, as a result of which, the tested composite sample was damaged. The obtained data, such as: tensile stresses, force at maximum load, the greatest tensile strain, displacement, and module, are included in the next table (Table 3).

**Figure 16:**
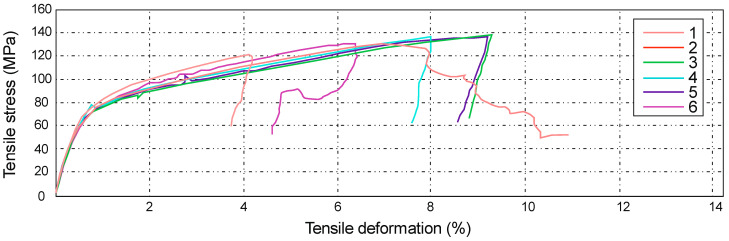
Diagram showing the tensile stress and absolute deformation of Roving IMS65 reinforced laminate, 0/90° configuration [31].

The figure below (Figure 16) shows the tensile tests results. In this case, during the strength test, the samples achieved different values of both elongation and strength. The maximum deformations fluctuated around 9%, and the minimum elongation was 4.21%, as a result of which, an average value of 7.32% was obtained, i.e., a displacement of 13.62 mm. The highest tensile stress value was 133 MPa and the smallest was 117 MPa, which corresponds to 7.98 kN and 6.41 kN, respectively.

In the load diagram, a clear change in its shear and slope can be seen. The appearance of such a linearity disturbance on the diagram indicates the accumulation of diffuse microcracks. They lead to the formation of damage that may affect the thickness of separate layers in the composite, which is synonymous with the beginning of irreversible damage to the material. The value for the tensile strength of Roving IMS65 reinforced composite laminate is $R_{m}$ = 128 ± 11 MPa.

**Figure 17:**
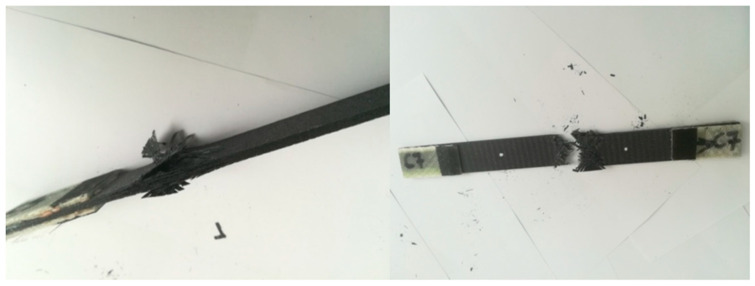
The appearance of samples from the C series after tensile bench testing.

In the C series, the composite was reinforced with the IMS65 CTLX biaxial fabric. As a result of the test, the fiber adhered to the matrix (Figure 17).

The samples were prepared in accordance with the ASTM D3039 standard, the dimensions of which are shown in Table 4. Additionally, the use of glass–epoxy caps allowed for mounting in the holders of the testing machine.

For all samples from the C series, a strength test was performed, until the structure of the composite material was destroyed. The results of the study were automatically implicated in a dedicated computer program (Table 5).

**Figure 18:**
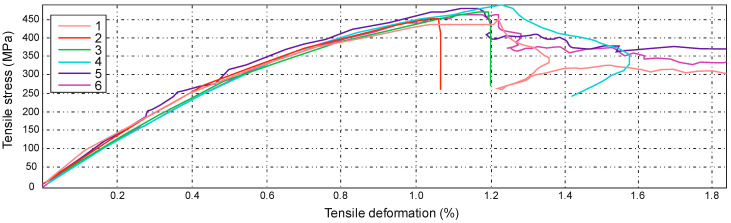
Graph showing the tensile stress and deformation of a laminate reinforced with IMS65 CTLX biaxial fabric, ± 45° configuration.

The next figure (Figure 18) illustrates the results of the tensile bench testing for series C, samples marked successively 1–6. Based on the above table, it can be concluded that the highest tensile force was 32.19 kN, which equals 526 MPa, while the lowest force value at maximum load was 29.54 kN, which corresponds to 512 kN. The samples were stretched at the level of about 5.32 mm. 

**Figure 19:**
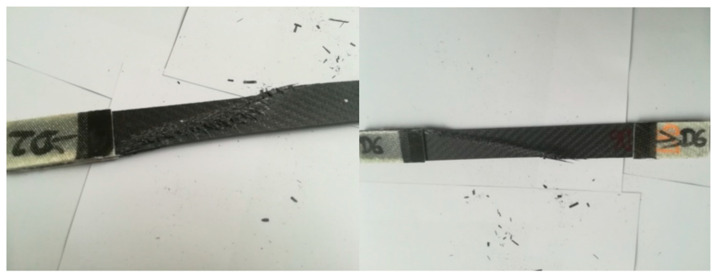
Appearance of samples from the D series after tensile bench testing.

The D series samples were made in accordance with the ASTM D3039 standard and reinforced with the symmetrical Interglas 02037 fabric. During the test, the sample was damaged, as shown in Figure 19.

The samples that were used in the test were cut from the board in accordance with the dimensions specified in the standard. Dimensions of individual samples are presented in the table above (Table 6).

Testing the composite material consisted of placing samples in the grips of the testing machine and subjecting them to a tensile test.

During the stretching process, the samples were gradually damaged. The tensile stresses, force at maximum load, the greatest tensile strain, displacement, and the modulus, with which the samples were loaded, are included in Table 7.

**Figure 20:**
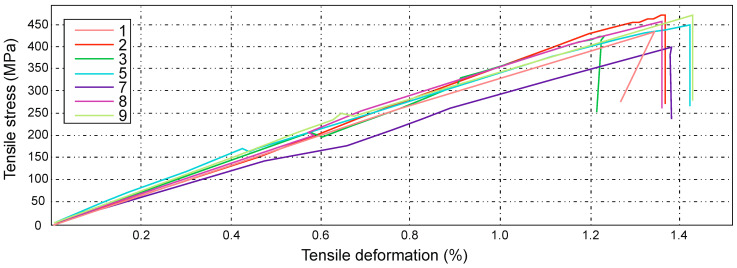
Graph showing the tensile stress and deformation of a laminate reinforced with a symmetrical Interglas 02037, 0/90° configuration.

The characteristics obtained for the D series (Figure 20) show the results of tensile strength tests for samples marked with numbers 1, 2, 3, 5, 7, 8, and 9.

The highest tensile stresses ranged from 457 MPa to 472 MPa, while the smallest differences were in the range from 414 MPa to 426 MPa. The average value of the tensile stress, in this case, is equal to 443 MPa, which is equal to the force value of 32.89 kN.

The highest tensile strain was close to the value of 1.37%, which is 5.43 mm. For the samples marked with numbers 2, 3, 7, 8, and 9, a sharp drop in the force value was noticed at the final stage of the strength test. This phenomenon is characterized by the so-called delamination of individual layers of composite laminate.

The tensile strength value of the composite laminate reinforced with the symmetrical Interglas 02037 fabric is equal to $R_{m}$ = 443 ± 29 MPa.

**Figure 21:**
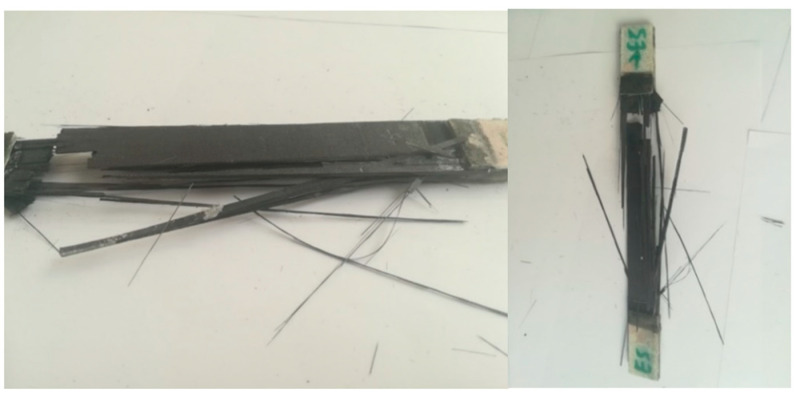
Appearance of samples from the E series after tensile bench testing.

The samples for the E series were made according to the guidelines contained in the ASTM D3039 standard. IMS65 modular fabric was used as reinforcement. The characteristic form of the tested material failure is shown in Figure 21.

The samples were cut from a uniform sheet of composite material. Their dimensions, which are presented in the table below (Table 8), are strictly regulated by the standard.

The test would not be possible without special overlays on the edges of the laminate, which allow the Instron testing machine to be placed in the holder.

The tensile strength test was carried out, which resulted in permanent damage to the structure of the composite laminate. The table below (Table 9) shows the most important parameters obtained during the tensile test.

**Figure 22:**
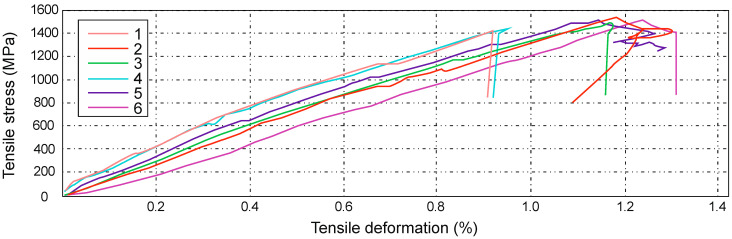
Graph showing the tensile stress and deformation of a laminate reinforced with IMS65 modular fabric, 0/90° configuration.

When analyzing the characteristics presented in the next figure (Figure 22), it can be noticed that the tensile stresses, during loading, for each sample were, on average, 1450 MPa. The sample elongation was about 1% at a comparable level.

On the basis of the analysis, one can also observe a linear increasing dependence of the tested samples, which proves that samples for the E series of composites reinforced with the IMS65 modular fabric behave like brittle materials during the strength test.

The table below (Table 10) presents the comparative characteristics of the averaged tensile strength $R_{m}$ (MPa) for all five series of tested samples. The strength test was designed to test various composite samples for their tensile strength. Strength tests were carried out on five different series of samples, with a different fiber arrangement and technology of their production.

Significant observations from the conducted tests and a comparative summary of the results obtained for different batches of materials are presented below [31].

Based on the results obtained, the following important observations can be presented:

1. The lowest value, in terms of tensile strength, was obtained for series B, which was R_m_ = 128 ± 11 MPa; the composite materials for this series were reinforced with a modular fabric of Dialead K63712 type with MGS L285/H285 warp, with 0/90° orientation.
2. In the case of testing the strength of the E series composite material, which determined its highest averaged value, the obtained results significantly differed from the results from the other series. For example, with regard to the Roving IMS65 reinforced composite material, its value was R_m_ = 1450 ± 70 MPa.
3. The tested sample No. 5 of series B (Table 3), which is a laminate of the Roving IMS65 reinforced composite material, with the 0/90° orientation, was characterized by the highest elongation (16.53 mm).
4. The tested sample No. 2 of series E (Table 9), which was a laminate of a composite material reinforced with the IMS65 modular fabric, with the 0/90° orientation, was characterized by the lowest elongation, and its tensile strain was 0.798%.

The next table (Table 11) shows the average hardness values, made using the Shore’s method. All samples were made by hand lamination using MGS L285/H285 epoxy resin. The hardness test was carried out maintaining the same laboratory conditions.

The composite reinforced with the IMS65 CTLX biaxial fabric was characterized by the highest hardness, the value being 85.0 Sh°D. However, the lowest hardness was obtained for the composite laminate reinforced with the symmetrical Interglas 02037 fabric, which was 77.3 Sh°D.

The following part of this article presents the proprietary FEM (finite element method) application for the strength analysis and optimization of the geometry of a sample composed of composites. The designed sample was modeled using SolidWorks and ANSYS environment software. The results of simulation strength tests for stresses, as well as the results of the calculations of deformations and displacements, occurring during the work of the sample are presented. 

## 3. The Results of Research Carried out in an Environment of ANSYS and SolidWorks

In the simulation tests carried out in the ANSYS environment, a GLARE 3 2/1-0.4 composite was used (a material composed of two aluminum sheets and two layers of prepregs). The aluminum layers were sheets with a thickness of 0.27 mm from the alloy 2024. 

The unidirectional layer of the prepreg, with a thickness of 0.27 mm, consisted of epoxy resin and long glass fibers of the R type (fiber diameter 15 μm), and the fiber content in the prepreg was approximately 64%. The thickness of the two prepreg layers (0/80) in the composite was 0.7 mm, and the total thickness of the GLARE 3 2/1 type composites was 1.4 mm.

**Figure 23:**
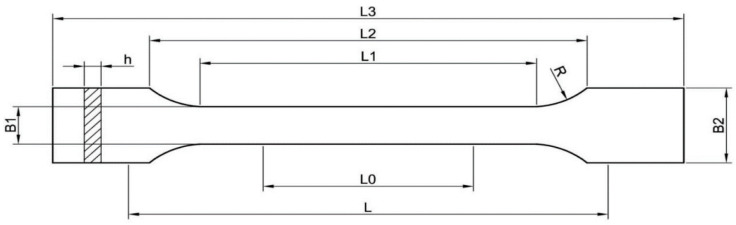
Universal moulder used in computer simulations.

The materials were made of one type of aluminum alloy and as the so-called mixed composites (e.g., 2024/prepreg/prepreg/2024). The order of the individual layers of the aluminum alloy sheet is shown in Figure 23. Table 12 shows the composition of the 2024 aluminum alloys, while the properties of the constituent materials of the composite are given in Table 13.

Then, on the basis of the mixing rule, the properties of the composites composed of various aluminum alloys were estimated, with a constant volume fraction and thickness of the composite layers.

The strain distributions ($\varepsilon_{x}$ and $\varepsilon_{y}$) in the samples were also analyzed using the finite element method. For this purpose, a 2D model was made in the design support system using the SolidWorks program (which, after converting to ANSYS, was simulated). The sample was modeled in such a way that it consisted of 4 layers, with a thickness of 0.25 mm each (thickness of the actual sample approximately 1 mm). This was to bring the model closer to the actual laminate, composed of four laminates of similar thickness and the same fiber directionality, for each of the layers.

Additionally, boundary conditions were imposed on the geometric model. All degrees of freedom were removed from the three edges of the tested sample, imposing a condition and assigning a value of zero to the appropriate components of the displacement vector at selected nodes. Regular partitioning was used to discretize the area of analysis, in order to obtain the best results of the calculations [36,37]. 

The direction of the fibers was defined by the appropriate orientation of the coordinate systems in the elements and assigning them the appropriate material constants. The calculations were based on the material data of the laminate, obtained experimentally and from the available data provided by the manufacturer of the materials (aluminum and prepreg).

### 3.1. Determination of the Interlayer Shear Strength of a Composite

Structural materials with a layered structure may deteriorate, as a result of delamination. Such a crack is formed inside the plate or shell element (made of a layer composite) and may locally, significantly reduce the flexural strength index and the bending stiffness.

The delamination created in the middle of the wall thickness, i.e., dividing the laminate into two sublaminates, reduces the bending strength index of the entire section at this point twice. Often this type of failure begins with the formation of a small delamination, due to the impact of a body (e.g., stone and tool). It then propagates according to the first or second method of loading (according to the nomenclature used in fracture mechanics).

In bars, plate and shell elements transmitting the bending moment and the transverse force, this type of delamination can cause tangential tensions, due to the transverse force. In large structural elements (e.g., a hull of a yacht), the delamination area may reach several square meters. One of the values characterizing the susceptibility of laminates to delamination is the strength to interlayer shear.

In the literature, on the subject of research, it is referred to as ILSS (interlaminar shear strength), or $\tau_{ILSS}$, characterizing the properties of the material subjected to transverse loads. Value $\tau_{ILSS}$ is one of the most important mechanical characteristics of polymer composites. The easiest way to determine the value $\tau_{ILSS}$ is to test a short beam by three-point bending (the term “stocky” is also used).

With the ratio of the support distance to the specimen height l/h = 5, the tangential tensions generated by the transverse force reach a value that causes the specimen to be destroyed by longitudinal shear at the height of the neutral bending layer. It happens before reaching the critical value of normal tension, σ, associated with the bending moment, which is expressed by the following inequality (1):

(1) $$\frac{\tau }{\tau_{ILSS}}>\frac{\sigma }{\sigma_{fM}}$$

There are many other methods of determining interlayer shear strength, but the short beam test is undoubtedly the most common, probably due to the simplicity of its implementation. A significant problem is the applicability of Żurawski’s formula to define tangential tensions in materials that are characterized by layered structure and the significant anisotropy of mechanical properties.

For these reasons, it is recognized that the values ($\tau_{ILSS}$) determined in the short beam bending test can be used for comparative assessment of materials selected for a specific application or new materials being developed in research institutes. However, they should not be used in the strength design of composite elements.

### 3.2. Tensile Test of the Composite GLARE 3 Type

The sample, called a specimen in the standard, is flat and has the shape of a “paddle” (Figure 23). The dimensions of the sample shall be as follows: thickness 4 ± 0.2 mm, measuring part width 10 + 0.2 mm, and an overall length over 150 mm.

In the case of directly formed specimens, the measuring part has a length of 80 + 2 mm (type A1) and in the case of a mechanically formed piece, 60 + 0.5 mm (type B1). Table 14 presents all dimensions of the B1 sample used in the tests.

The moulder was modeled in the SolidWorks program, in which a file in the STL format, necessary to create the machine code, was created. In the STL format, the surface of the model was approximated with a network of triangles. The file contained the coordinates x, y, and z of each triangle vertex and a vector normal to the surface of each triangle. Such a record causes inaccuracies in the representation of the model, and the created triangles do not perfectly reflect the real surface. The more triangles, the greater the accuracy, but also, the larger the file size. Based on the STL file in the Cura program, the machine code was created, the so-called G-code.

Each composite layer was modeled with at least two layers of rectangular or hexagonal elements. The polymer composite was treated as a material with orthotropic properties, and the duralumin was treated as an elastic–plastic material with strengthening, with properties described by the curve σ=σ(ε) and Poisson’s ratio of 0.3. All calculations were performed in the SolidWorks program.

**Figure 24:**
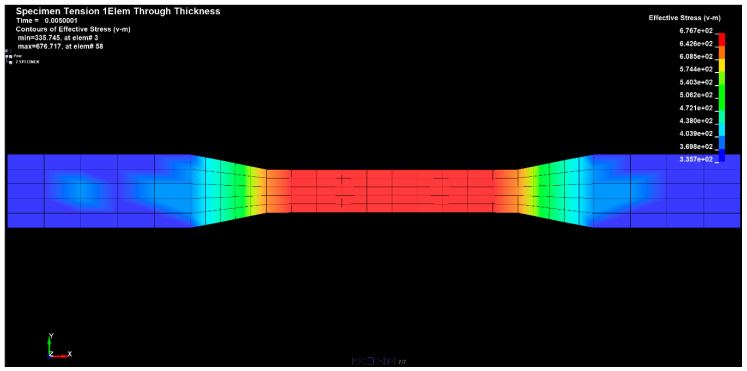
Sample tension in the x direction.

**Figure 25:**
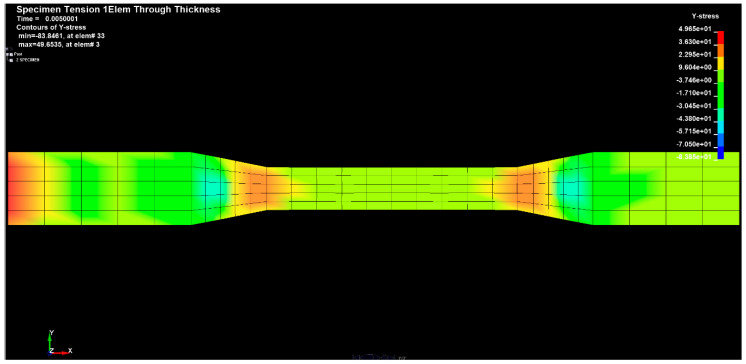
Sample tension in the y direction.

**Figure 26:**
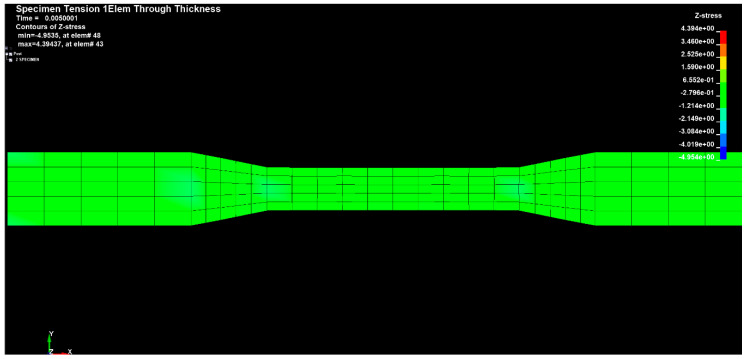
Sample tension in the z direction.

Figure 24, Figure 25 and Figure 26 show the diagrams of the tensions in the directions x, y and z of the stretched sample, obtained by the finite element method in the SolidWorks program. The moulders were stretched with a longitudinal force of 1200 N. It can be seen in the diagrams, that when the reinforced fibers were added in the prepreg in the direction x, the minimum tension value was 335.745 (Pa), and the maximum value was 676.717 (Pa).

The results for the direction y show that the tension range was in the range from −83.848 (Pa) to 49.6535 (Pa).

For the sample tension in the direction z, the range of the minimum and maximum values ranged from 335.745 (Pa) to 676.717 (Pa). All the assessed values of the ARI (adhesive remnant index) index in the discussed group of samples were characterized by the presence of mixed adhesive–cohesive fractures, both within the material and on the profile of a given sample.

**Figure 27:**
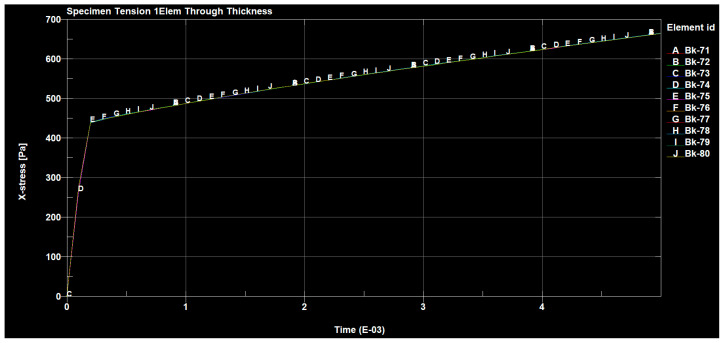
Sample deformation in the x direction.

**Figure 28:**
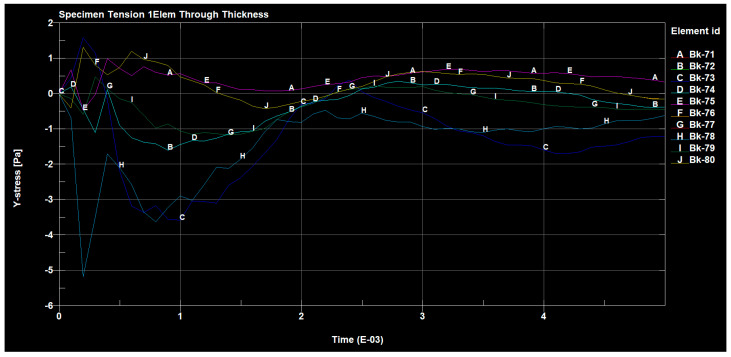
Sample deformation in the y direction.

**Figure 29:**
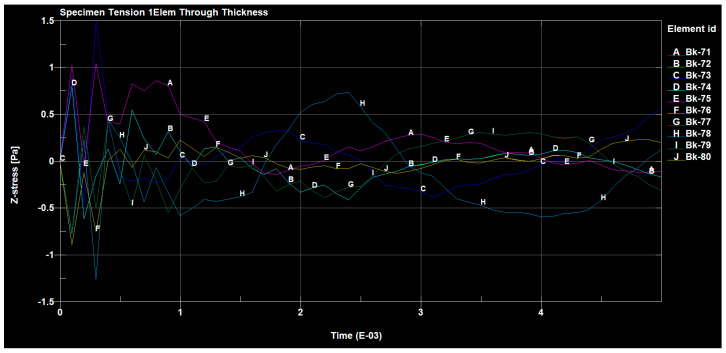
Sample deformation in the z direction.

The selected results of the tension strain waveforms of the analyzed sample, using the finite element method for a series of preliminary tests using the clamping force, are presented in the figures below (Figure 27, Figure 28 and Figure 29).

**Figure 30:**
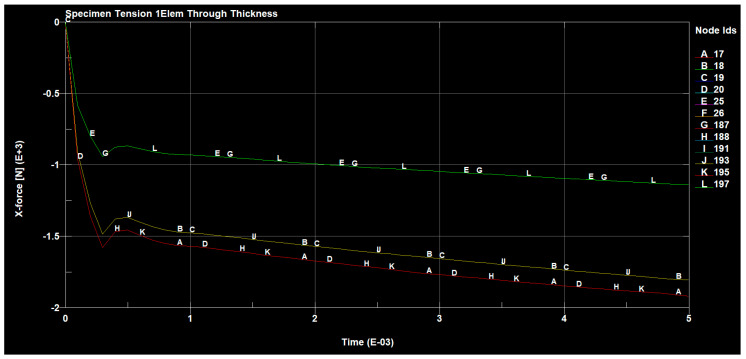
Tensile forces occurring on the sample in the x direction.

**Figure 31:**
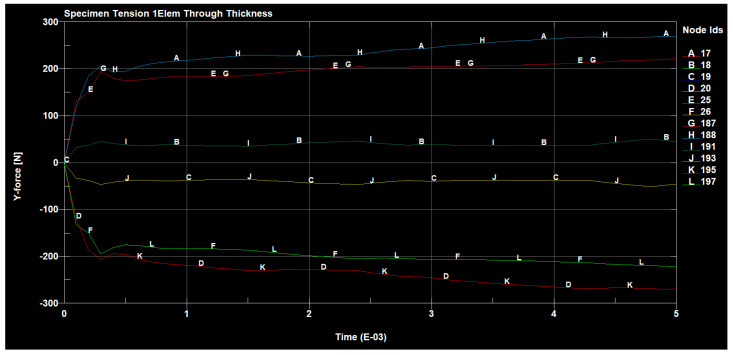
Tensile forces occurring on the sample in the y direction.

**Figure 32:**
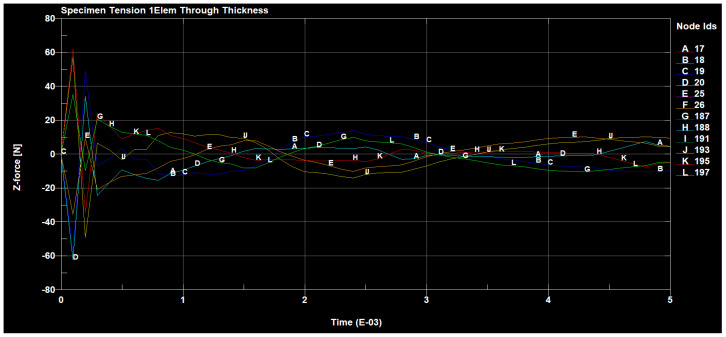
Tensile forces occurring on the sample in the z direction.

The tangential tension values, presented in Figure 30, Figure 31 and Figure 32, contained within the limits (700 MPa) obtained in the shear test, confirmed the good adhesive properties of the bonding system. The analysis of the fracture planes showed that, in the conducted study, only mixed fractures of an adhesive–cohesive nature occurred.

**Figure 33:**
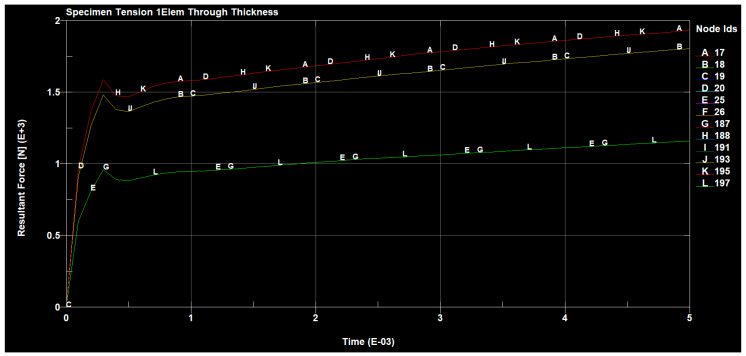
Total tensile forces present in the sample.

The forces occurring, in the tested sample, in all directions are shown in the following figures (Figure 30, Figure 31, Figure 32 and Figure 33).

The value of the applied forces was selected so that the normal stresses in the direction x reached the values of the yield point of the alloy in the outer aluminum layers of the tested material.

The values of the normal stresses perpendicular to the composite layers and the tangential stresses between these layers, calculated in the most loaded cross-section (located in the central part of the model), turned out to be negligible, compared to the normal stresses in the direction x, which were higher in the aluminum layers. In such a loaded sample of the layered composite, with the fiber in the area outside the grips, not only normal stresses $\sigma_{x}$ occur but also $\sigma_{y}$, which can cause delamination and tangential $T_{xy}$ stresses that can cause interlayer shear.

Based on the simulations carried out, it was found that at lower load values, to a greater extent, are transferred through the aluminum layers and after the metal yield point is exceeded by the layers of the composite with additional fiber. At the breaking load of the tested composite material, the stress value in the composite was close to its resistance determined experimentally. On the other hand, increasing the strength of the composite with the fiber component should increase the strength of the tested material.

Tensile and bending strength tests of laminates are subject to similar conditions as tests of unidirectional composites. Problems may arise in the bending and tensile tests of samples cut in directions not coinciding with the principal directions of the stress state. Such samples tend to change shape under load by losing their flatness. The limitation of the possibility of displacement by the fastening parts in the testing machine constitutes a factor disturbing the course of the tensile and bending tests of samples cut in directions that are not the main directions of the anisotropy of the elastic properties of the material. As a result, larger measurement errors of given quantities are obtained.

Measurements of the strength properties were made by static tensile tests using the ANSYS software, in the scope of the strength test in the measuring range of the tensile force with the value of 1200 N in class 1. For the samples prepared in this way, a static tensile test was carried out, thanks to which, information about the minimum local properties was obtained over a section equal to the base of measuring sample.

The performed numerical calculations revealed that the nonlinear strain–stress characteristics for a composite with an additional fiber compressed across the fibers strongly depended on the strength properties of the interphase and the adhesion forces at the fiber–interphase interface.

## 4. Summary and Conclusions

The conducted research process of composite materials, used in their structure reinforcements in the form of polymer fibers, indicates that materials of this type require constant evaluation and further exploration in the field of more and more perfect and newer construction materials; in particular, those related to the issues of material strength, e.g., in terms of vibration damping, lightness, or resistance to external factors, which have had a key impact on their practical application in the aviation industry.

In this aspect, it should be noted that the choice of material has a significant impact on the quality and reliability of aircraft structures, which are the basic factors enabling progress and indicating further directions of development. Aircraft structures have special requirements in this regard; specifically, materials that are lightweight, mechanically resistant, and corrosion resistant are searched for.

An important aspect in the modern design of aircraft structures is the FEM method and the software that uses it, including the ANSYS and SolidWorks environment used in this article. Computer programs allow you to determine the weaknesses of the structure at a low cost, as well as to calculate both the service life and serviceability of aviation equipment. One of the directions of the development of materials used in aviation structures will undoubtedly be hybrid or composite-metal materials. It turns out that despite their high production costs, very favorable mechanical properties can be achieved, and sometimes the undesirable effects that are typical for them can be prevented.

An example is the material of aluminum with a composite type, which, thanks to the aluminum–composite structure, prevents delamination and strongly propagating cracks. In the aviation industry, one should expect continuous competition between metal and composite materials, thus setting a new direction in designing materials for aviation, the aim of which is to achieve 20–30% lower weight and 20–40% lower manufacturing costs. In this regard, intensive research is carried out all the time to develop new materials and technologies for aviation applications, enabling the improvement of aircraft design.

The article presents selected and currently developed methods of assessing the strength of composite structures, where each of the applied methods of nondestructive diagnostics has both its limitations and possibilities, depending on the tested material, shape, or thickness of joints. Currently, from a wide group of functional materials, the most important in aviation applications are laminates containing polymer-matrix composites and composites with built-in intelligent elements for monitoring the condition of the structure and for controlling its properties.

The results of the strength analysis show that the elements most vulnerable to damage are constrictions on the sample, because the constriction directly contributes to the deterioration of its material strength. Subsequently, the sample was subjected to a strength analysis under the same boundary and initial conditions.

From a series of solutions, the variant with the lowest maximum values was selected for the displacement, deformation, and stresses. This variant was transformed into the ANSYS environment, in order to optimize the layered structure of the sample, where the optimization criterion were to minimize the values of displacements, stresses, and strains. The program generated three optimal solutions, among which the variant with the lowest possible values of the output variables was selected.

It should be noted that the use of software supporting engineering design greatly facilitates the work of a potential engineer. Without the use of FEM software, the problem posed in this paper would be much more time-consuming and solved with significant simplification.

Moreover, by using the simulation software, time can be significantly reduced; additionally, the costs of designing new devices can be minimized. Nowadays, costly prototype tests are often replaced by simulation and optimization tests using the FEM method, SolidWorks, etc.

It should be noted that the use of intelligent materials, based on composites and laminates, in the construction of aviation structures is one of the tasks that requires close cooperation between the research and development centers and aviation plants to make these plants competitive in the modern market.

## Figures and Tables

**Table 1:** Parameters of fabrics used as reinforcement of composites.

Parameter	Dialead K63712 Modular Fabric	Roving MS65	IMS65 CTLX Biaxial Fabric	Symmetrical Fabric Interglas 02037	IMS65 Modular Fabric
Fabric sheet density (g/m^2^)	41.9	32.3	66.8	47.5	27.2
Thickness (mm)	0.20	0.20	0.19	0.21	0.2
Tensile strength (MPa)	419	128	513	443	1450

**Table 2:** Geometric dimensions of Roving IMS65 reinforced composite samples.

The Numbers of the Samples	Width (mm)	Thickness (mm)	Area (mm^2^)	Length (mm)
1.	25.2	2.18	55.0	150
2.	25.2	2.36	59.4	150
3.	25.2	2.38	59.9	150
4.	25.6	2.34	60.0	150
5.	25.2	2.39	60.1	150
6.	25.1	2.31	58.0	150
Average	25.2	2.33	58.7	150

**Table 3:** Analysis of the obtained test results for the Roving IMS65 composite reinforced laminate, 0/90° configuration.

The Numbers of the Samples	Tensile Stress (MPa)	Maximum Force (kN)	The greatest Tensile Deformation (%)	Maximum Displacement (mm)	Module (MPa)
1.	117	6.41	4.21	8.99	13,200
2.	127	7.56	7.14	12.77	12,300
3.	133	7.98	9.11	16.18	12,500
4.	131	7.85	7.98	15.27	12,400
5.	133	7.98	9.22	16.53	12,200
6.	127	7.35	6.26	12.00	12,000
Average	128	7.52	7.32	13.62	12,400

**Table 4:** Geometric dimensions of composite samples reinforced with the IMS65 CTLX biaxial fabric.

The Numbers of the Samples	Width (mm)	Thickness (mm)	Area (mm^2^)	Length (mm)
1.	25.1	2.30	57.8	150
2.	25.2	2.49	62.7	150
3.	25.2	2.49	62.7	150
4.	25.1	2.32	58.3	150
5.	25.2	2.42	61.0	150
6.	25.2	2.42	60.9	150
Average	25.2	2.41	60.5	150

**Table 5:** Analysis of the obtained test results for the composite laminate reinforced with the IMS65 CTLX biaxial fabric, weave ± 45°.

The Numbers of the Samples	Tensile Stress (MPa)	Maximum Force (kN)	The Greatest Tensile Deformation (%)	Maximum Displacement (mm)	Module (MPa)
1.	512	29.54	1.17	6.24	58,300
2.	484	30.35	1.31	4.18	62,000
3.	514	32.19	1.25	4.63	56,900
4.	533	31.05	1.32	7.20	53,500
5.	526	32.12	1.24	5.02	63,400
6.	510	31.06	1.26	4.66	54,900
Average	513	31.05	1.26	5.32	58,200

**Table 6:** Geometric dimensions of composite samples reinforced with the symmetrical Interglas 02037 fabric.

The Numbers of the Samples	Width (mm)	Thickness (mm)	Area (mm^2^)	Length (mm)
1.	25.4	2.84	72.3	150
2.	25.3	2.94	74.4	150
3.	25.3	2.99	75.6	150
5.	25.5	3.01	76.6	150
7.	25.5	3.00	76.4	150
8.	25.3	2.87	72.6	150
9.	25.4	2.84	72.0	150
Average	25.4	2.93	74.3	150

**Table 7:** Analysis of the obtained test results for the composite laminate reinforced with the symmetrical Interglas 02037 fabric, 0/90° configuration.

The Numbers of the Samples	Tensile Stress (MPa)	Maximum Force (kN)	The greatest Tensile Deformation (%)	Maximum Displacement (mm)	Module (MPa)
1.	467	33.78	1.39	5.36	37,300
2.	447	33.28	1.37	4.65	36,000
3.	426	32.21	1.23	5.19	36,300
5.	419	32.14	1.43	5.27	32,700
7.	414	31.63	1.37	4.93	37,900
8.	456	33.14	1.37	5.47	37,500
9.	472	34.02	1.41	7.09	37,700
Average	443	32.89	1.37	5.43	36,500

**Table 8:** Geometric dimensions of composite samples reinforced with the IMS65 modular fabric.

The Numbers of the Samples	Width (mm)	Thickness (mm)	Area (mm^2^)	Length (mm)
1.	25.2	2.38	60.0	150
2.	25.2	2.48	62.5	150
3.	25.2	2.47	62.4	150
4.	25.3	2.44	61.6	150
5.	25.3	2.46	62.1	150
6.	25.3	2.425	61.9	150
Average	25.2	2.45	61.8	150

**Table 9:** Analysis of the obtained test results for a composite laminate reinforced with IMS65 modular fabric, 0/90° configuration.

The Numbers of the Samples	Tensile Stress (MPa)	Maximum Force (kN)	The Greatest Tensile Deformation (%)	Maximum Displacement (mm)	Module (MPa)
1.	1490	89.65	1.03	10.63	161,000
2.	1380	86.42	0.798	11.94	202,000
3.	1450	90.15	1.02	11.89	176,000
4.	1400	86.35	0.823	10.20	202,000
5.	1480	91.71	1.000	11.33	191,000
6.	1480	91.32	1.08	11.72	131,000
Average	1450	89.27	0.959	11.29	177,000

**Table 10:** Tensile strength R_m_ of composite laminates, reprinted from [31].

Designation of the Series	Tensile Strength R_m_ (MPa)	
A	419	±18
B	128	±11
C	513	±29
D	443	±29
E	1450	±70

**Table 11:** Results of hardness measurements, made by the Shore’s method.

Series	Name	Weave	Sh°D
A	Modular fabric Dialead K63712	0/+90°	81.6
B	Roving IMS65	0/+90°	80.2
C	IMS65 CTLX biaxial fabric	+/−45°	85.0
D	Symmetrical fabric Interglas 02037	0/+90°	77.3
E	IMS65 modular fabric	0/+90°	83.5

**Table 12:** Composition of aluminum alloys 2024.

Aluminium Alloy	2024	
Composition	Cu	4.6
	Mg	1.4
	Mn	0.54
	Fe	0.18
	Zn	0.1
	Si	0.09
	Ti	0.02
	Cr	0.01
	rest	0.05

**Table 13:** The properties of the constituent materials of the composite.

Material Type	Properties		
	Density (g/cm^3^)	E (GPa)	R_m_ (MPa)
2024	2.79	73	458

**Table 14:** Moulder dimensions.

Moulder Dimensions	Type B1 (mm)
L3—total length	150
L1—the length of the part delimited by lines	40
R—radius	60
L2—length between wide parallel parts	106
B2—width at the ends	20
B1—width of the narrow part	10
H—recommended thickness	4
L0—measuring length	50
L—initial distance between the handles	115

## Data Availability

The data presented in this study are available on request from the corresponding author.
